# Novel Use of a Soehendra Biliary Dilatation Catheter and Controlled Radial Expansion Balloon To Gain Access Through an Existing Duodenal Stent via Complex Endoscopic Retrograde Cholangiopancreatography: A Case Report

**DOI:** 10.7759/cureus.98358

**Published:** 2025-12-03

**Authors:** Rida Abbasi, Mohammed Korani, Javaid Iqbal, Venkata Lekharaju, Daniyal Baig, Sajjad Mahmood

**Affiliations:** 1 Gastroenterology, Wythenshawe Hospital, Manchester University NHS Foundation Trust, Manchester, GBR; 2 Gastroenterology and Hepatology, Wythenshawe Hospital, Manchester University NHS Foundation Trust, Manchester, GBR

**Keywords:** controlled radial expansion (cre) balloon dilation, duodenal stricture, ercp, malignant biliary obstruction, pancreatic cancer, pancreatobiliary, soehendra biliary dilatation catheter

## Abstract

We report the case of a malignant type II duodenal stricture treated with an uncovered duodenal stent, later requiring endoscopic retrograde cholangiopancreatography (ERCP) for biliary obstruction. The ampulla was covered with a stent mesh. The procedure was performed through the meshed duodenal stent to access the bile duct. In our case, we used a Soehendra Biliary Dilatation Catheter (Cook Medical®, Limerick, Ireland) and controlled radial expansion (CRE) to facilitate the passage of a fully covered self-expandable metal stent through the mesh. This technique has not been previously described in the literature. It demonstrates the feasibility of accessing malignant strictures in palliated patients by the use of a CRE balloon and a Soehendra Biliary Dilatation Catheter to widen the uncovered duodenal stent mesh in complex ERCP cases.

## Introduction

Malignant biliary obstruction (MBO) causing jaundice can be challenging to manage [1]. Commonly caused by pancreatic adenocarcinoma or cholangiocarcinoma, biliary drainage is recommended to decompress the biliary tree for symptomatic improvement, facilitating the progression of treatment to the next stage of management [2]. Endoscopic retrograde cholangiopancreatography (ERCP) with stent insertion is well established and the preferred option, with success rates of around 90% [3,4]. Additionally, approximately 15-20% of patients with pancreatic cancers develop gastric outlet obstruction [5,6], often presenting later in the disease course. Duodenal stenting is a safe and effective procedure that improves symptoms of gastric outlet obstruction, enabling better nutrition, early discharge from the hospital, and possibly improved survival by allowing for chemotherapy [7,8]. The majority of patients present with jaundice first and then progress at a later stage to develop duodenal obstruction. Therefore, ERCP would have been performed in these patients, establishing adequate biliary drainage, making duodenal stent insertion the next step. Here, we report a case where the patient underwent duodenal stent insertion for a malignant duodenal stricture and later developed jaundice due to MBO requiring biliary drainage.

ERCP through previously placed duodenal stents has been described in the literature. It is a challenging procedure, particularly in type II obstruction [9,10]. Such complex procedures are best performed in centers performing high volumes of ERCP [11,12]. Usage of either rat-tooth forceps or argon plasma coagulation for the removal or melting of wires of the stent’s mesh has been reported. In our case, we used a controlled radial expansion (CRE) balloon and Soehendra Biliary Dilatation Catheter (Cook Medical®, Limerick, Ireland) to dilate the stent’s mesh to facilitate the passage of a fully covered self-expandable metal stent (FcSEMS).

## Case presentation

**Figure 1:**
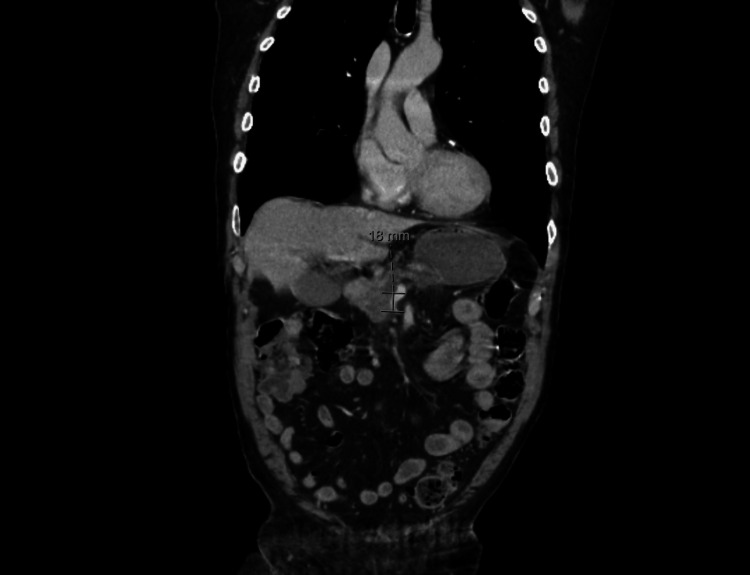
CT of the abdomen and pelvis showing a 4 cm mass on the pancreatic head abutting the superior mesenteric vein.

**Figure 2:**
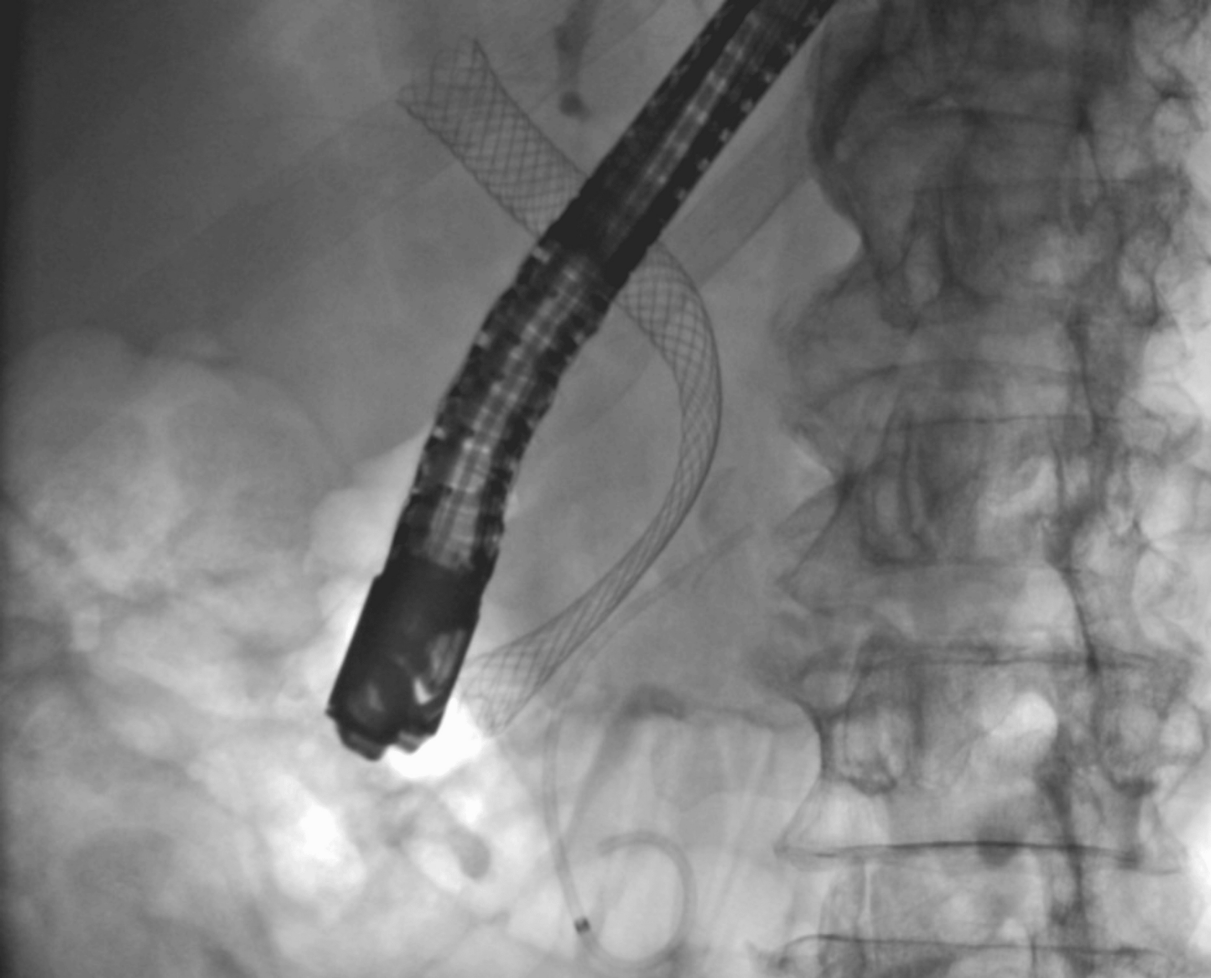
Malignant biliary stricture managed by insertion of a covered metal biliary stent.

A 77-year-old male presented with painless obstructive jaundice. A CT scan (anteroposterior) revealed a 4 cm pancreatic head mass abutting the superior mesenteric vein (Figure 1), diagnosed as locally advanced pancreatic cancer. On his first ERCP, there was distortion and stricturing of the duodenum, only passable with a cannula and wire for guidance. The ampulla was floppy and distorted; bile duct cannulation was only successful after pancreatic duct stenting and needle knife sphincterotomy. Cholangiogram showed a long, irregular stricture requiring fully covered metal biliary stents across the biliary stricture (Figure 2).

**Figure 3:**
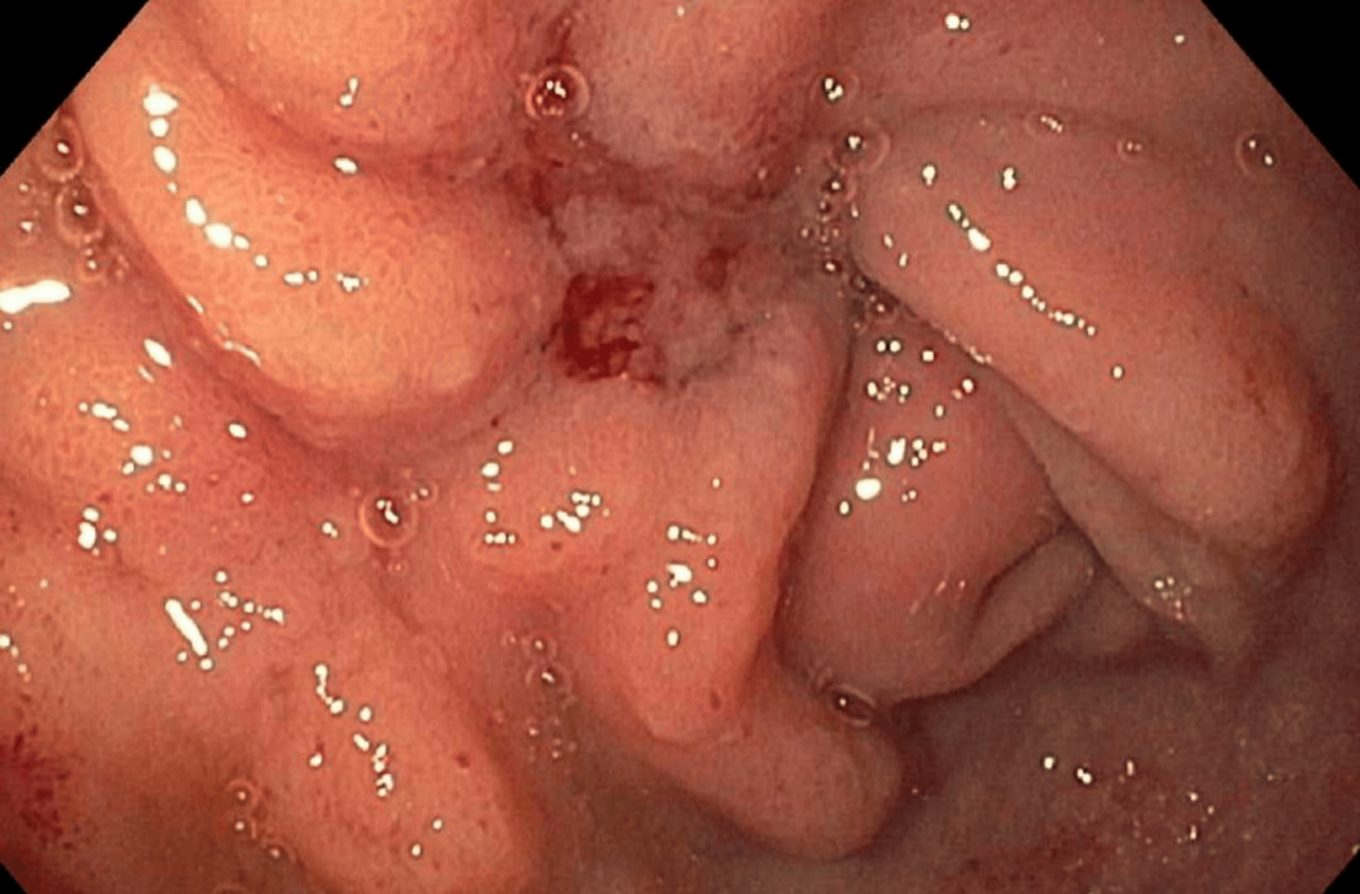
Esophagogastroduodenoscopy image of a type II, extrinsic, malignant-appearing duodenal stricture not traversable by endoscopy.

**Figure 4:**
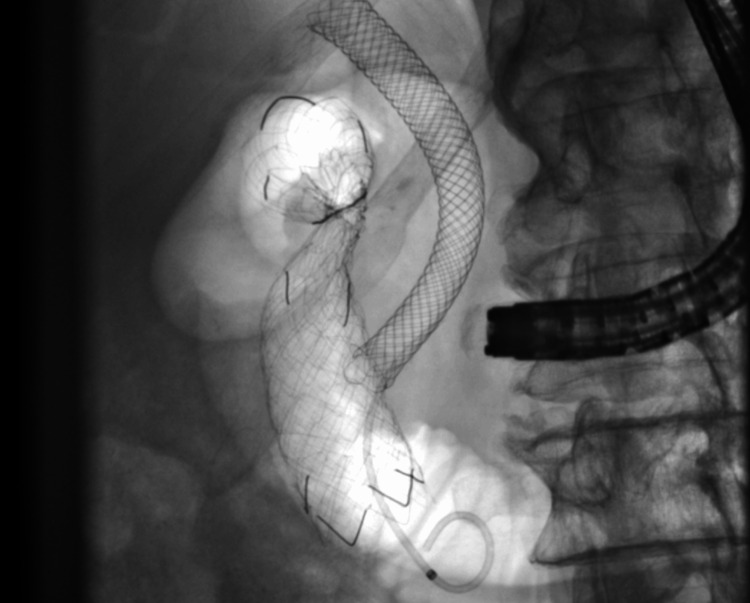
Uncovered self-expandable metal duodenal stent: fully covered self-expanding metal stent in situ.

Two months later, the patient developed symptoms of gastric outlet obstruction. Esophagogastroduodenoscopy (EGD) revealed a type II [9,10] extrinsic malignant appearing duodenal stricture not traversable by endoscopy (Figure 3). An uncovered self-expandable duodenal stent was placed using fluoroscopy and a guide-wire in another hospital (Figure 4). (All figures were free to use.)

**Figure 5:**
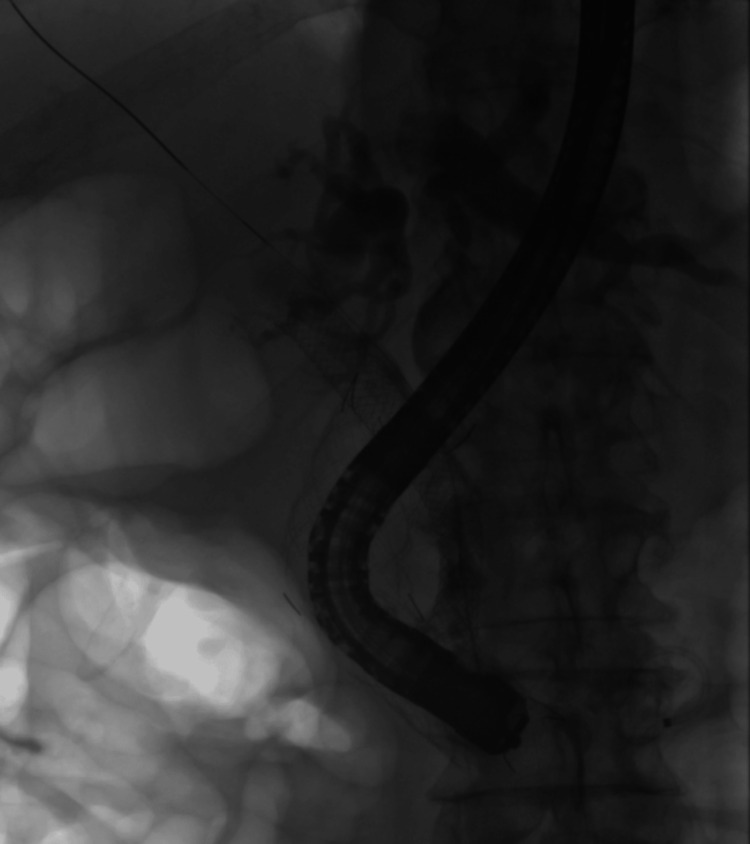
Soehendra catheter passed through the duodenal stent into the biliary stent.

**Figure 6:**
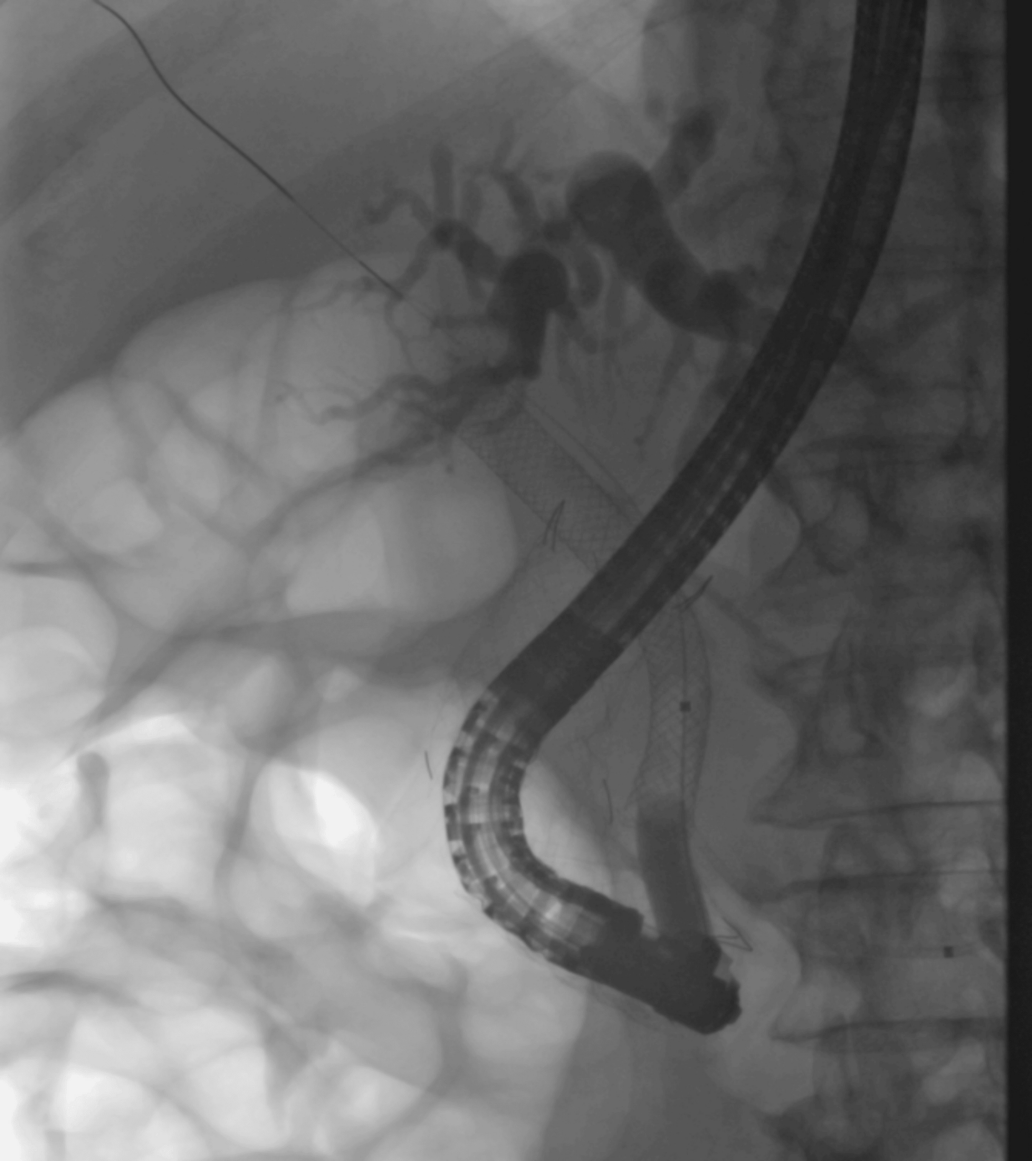
Controlled radial expansion balloon to dilate the duodenal stent to insert a fully covered self-expandable metal stent.

Nine months later, the patient presented with cholangitis and recurrent jaundice. His second ERCP was technically challenging as the previously placed uncovered duodenal stent covered the ampullary orifice. The ampulla was difficult to visualize due to tumor ingrowth and accumulated food debris within the stent, and the exact location was guided by the prior pancreatic duct and biliary stents under fluoroscopy. Cannulation was achieved by passing a sphincterotome through the mesh of the duodenal stent into the existing biliary stent. Multiple balloon travels removed debris and sludge. As passage of FcSEMS through the duodenal stent mesh was not possible, we used a Soehendra Biliary Dilatation Catheter (Figure 5) and an 8 mm CRE balloon to dilate the mesh opening (Figure 6), allowing placement of an additional FcSEMS, with the distal end within the duodenal stent, to improve biliary drainage and resolve sepsis.

## Discussion

We present a complex ERCP case of a pancreatic cancer patient who previously had biliary and duodenal stents inserted in another hospital. Identifying the obscured papilla through the duodenal stent mesh poses challenges​ [10-12]. Typically, wires of the mesh may be removed with forceps or melted using argon plasma coagulation if new mid-portion lattice duodenal stents were not used​ [10,11]. However, in our case, we successfully used a Soehendra biliary dilator and a CRE balloon to navigate a new FcSEMS through the duodenal stent mesh. The Soehendra biliary dilator is a well-known tool used to dilate biliary and pancreatic strictures where other accessories are not applicable. Of note, 10-20% of patients with pancreatic cancers who require biliary stenting also have duodenal obstruction​ [8]. Our case highlights an innovative approach to a common dilemma using widely available, familiar, well-known accessories to avoid invasive alternatives such as percutaneous transhepatic cholangiography or endoscopic ultrasound-guided biliary draining.

## Conclusions

In this case, the use of a Soehendra biliary dilator and CRE balloon in the case of head of pancreas cancer, which caused a malignant biliary stricture and gastric outflow obstruction, has been demonstrated. The management of both stricture and obstruction was performed by the insertion of a self-expanding metal stent, which is an effective palliative treatment for such conditions. The patient had a recurrence of biliary blockage, which was addressed by these common tools in an innovative manner. Re-access to the duodenal stent via insertion of FcSEMS to maintain biliary drainage was successful. This novel approach highlights an opportunity to address malignant strictures in palliated patients in complex ERCP cases.
